# Reply to Siniorakis et al., “COVID-19 Interference with Renin-Angiotensin System in the Context of Heart Failure”

**DOI:** 10.1128/mBio.01243-20

**Published:** 2020-05-29

**Authors:** David S. Fedson, Steven M. Opal, Ole Martin Rordam

**Affiliations:** a 57, chemin du Lavoir, Sergy Haut, France; b Department of Medicine, Alpert Medical School of Brown University, Providence, Rhode Island, USA; c Fyordgata 59, Trondheim, Norway

**Keywords:** angiotensin receptor blockers, COVID-19, statins, angiotensin converting enzyme inhibitors, neprilysin inhibitors

## REPLY

We thank Eftychios Siniorakis and colleagues for their thoughtful letter on how repurposing statins and angiotensin receptor blockers (ARBs) for coronavirus disease 2019 (COVID-19) treatment might affect patients with heart failure (1). Our understanding of the pathophysiology of COVID-19 disease has advanced rapidly in the past few months. The immunological dysregulation associated with the disease and the cardiovascular effects of infection (and especially the role of angiotensin converting enzyme 2 [ACE2]) have been comprehensively reviewed (2–7). Some patients who develop acute respiratory distress syndrome (ARDS) can be severely hypoxic and yet show relatively normal pulmonary compliance (8). Endothelial dysfunction and intense inflammation seem to be important contributors to their distress (9). In addition, pulmonary microvascular coagulopathy, sometimes associated with pulmonary or systemic embolization, has added a new dimension to clinical care (10–12). Many physicians have added anticoagulation to their treatments (13). Recently, van de Veerdonk and colleagues called attention to the contribution of the kallikrein-kinin system to COVID-19-induced ARDS (14, 15). Although much attention has been focused on the relationship between the renin-angiotensin system (RAS) and COVID-19 (5–7), there is considerable cross talk between the RAS and kallikrein-kinin systems (14–16). Experimentally, a reduction in ACE2 activity can impair inactivation of the B1 bradykinin receptor and this can be associated with an increase in inflammation-induced acute lung injury (17).

This is the backdrop for the concern raised by Siniorakis et al.: some COVID-19 patients might have heart failure and be receiving combination treatment with an angiotensin receptor blocker (ARB)/angiotensin receptor neprilysin inhibitor (ARNI). Neprilysin is known to degrade bradykinin, and ARB treatment can increase bradykinin levels (18). Thus, if bradykinin is involved in the genesis of the intense inflammation and microvascular coagulopathy seen in many COVID-19 patients (11, 14, 15), the increase in bradykinin levels that accompanies ARB and neprilyin inhibitor treatment might be harmful. (Angiotensin converting enzyme inhibitors [ACEIs] also upregulate bradykinin, and they are more commonly associated with acute episodes of angioedema than are ARBs.) To our knowledge, there have been no reports of COVID-19 patients who were treated with both ARNIs and ARBs or ACEIs.

We believe that physicians should consider combination statin/ARB treatment of severely ill COVID-19 patients (19). Both drugs have beneficial effects on inflammation, coagulation abnormalities, and endothelial dysfunction. Recently published observational studies suggested that ACEIs, ARBs, and statins are associated with improved outcomes in COVID-19 patients (20, 21), although not all studies have shown this (22). Whether ARNI treatment of COVID-19 patients would be harmful remains to be determined, although it has been shown to improve endothelial dysfunction in hypertensive rats (23). The great advantage of ACEIs, ARBs, and statins is that they are widely available as inexpensive generics in resource-poor countries where lockdowns and social distancing would be difficult to implement. The same cannot be said for virtually all of the other COVID-19 treatments now being tested in clinical trials.
